# The *AP5B1* p.Leu785Pro variant is a frequent cause of late-onset macular dystrophy with variable extraocular manifestations

**DOI:** 10.1016/j.xhgg.2026.100659

**Published:** 2026-08-07

**Authors:** Petra Liskova, Lubica Dudakova, Karolina Kaminska, Laura Kühlewein, Stefanida Shliaga, Rina Leibu, Miriam Ehrenberg, Dinah Zur, Jana Zernant, Miriam Bauwens, Gavin Arno, Isabelle Müller, Focke Ziemssen, Viktoria Bothe, Francesca Cancellieri, Julie De Zaeytijd, Pascale Mazzola, Winston Lee, Bohdan Kousal, Marie Vajter, L. Ingeborgh van den Born, Anton Sonntag, Yoeri van Leeuwen, Dzenita Smailhodzic, Caroline C.W. Klaver, Tobias B. Haack, Bernd Wissinger, Andrew R. Webster, Lonneke Haer-Wigman, Siying Lin, Moreno Menghini, Konrad Platzer, Elfride De Baere, Rando Allikmets, Carlo Rivolta, Susanne Roosing, Susanne Kohl, Tamar Ben-Yosef, Mathieu Quinodoz

**Affiliations:** 1 Department of Paediatrics and Inherited Metabolic Disorders, First Faculty of Medicine, Charles University and General University Hospital in Prague, 128 08 Prague, Czech Republic; 2 Department of Ophthalmology, First Faculty of Medicine, Charles University and General University Hospital in Prague, 120 00 Prague, Czech Republic; 3 Institute of Molecular and Clinical Ophthalmology Basel (IOB), 4031 Basel, Switzerland; 4 Department of Ophthalmology, University of Basel, 4031 Basel, Switzerland; 5 Centre for Ophthalmology, University Eye Hospital, University Hospital Tübingen, 72076 Tübingen, Germany; 6 Department of Human Genetics, Radboud University Medical Center, Nijmegen 6525 GA, the Netherlands; 7 Department of Ophthalmology, Rambam Health Care Campus, Haifa 3109601, Israel; 8 Department of Ophthalmology, Schneider Children’s Medical Center of Israel, Petah Tikva 4920235, Israel; 9 Division of Ophthalmology, Tel Aviv Sourasky University Medical Center, Tel Aviv 6423906, Israel; 10 Gray Faculty of Medical & Health Sciences, Tel Aviv University, Tel Aviv 6997801, Israel; 11 Department of Ophthalmology, Columbia University, New York, NY 10027, USA; 12 Center for Medical Genetics, Ghent University Hospital, 9000 Ghent, Belgium; 13 Department of Biomolecular Medicine, Ghent University, 9000 Ghent, Belgium; 14 JC Self Research Institute, Greenwood Genetic Center, Greenwood, SC 29646, USA; 15 UCL Institute of Ophthalmology, University College London, London EC1V 9EL, UK; 16 National Institute of Health Research Biomedical Research Centre, Moorfields Eye Hospital, London EC1V 9EL, UK; 17 Eye Clinic, Luzerner Kantonsspital (LUKS), 6004 Luzern, Switzerland; 18 Institute of Human Genetics, University of Leipzig Medical Center, 04103 Leipzig, Germany; 19 Department of Ophthalmology, Ghent University Hospital, 9000 Ghent, Belgium; 20 Institute of Medical Genetics and Applied Genomics, University of Tübingen, 72076 Tübingen, Germany; 21 Bascom Palmer Eye Institute, University of Miami Miller School of Medicine, Miami, FL 33136, USA; 22 The Rotterdam Eye Hospital, Rotterdam 3011 BH, the Netherlands; 23 Department of Ophthalmology, Radboud University Medical Center, Nijmege 6525 GA, the Netherlands; 24 Department of Ophthalmology, Erasmus Medical Center Rotterdam, Rotterdam 3015CE, the Netherlands; 25 Department of Epidemiology, Erasmus Medical Center Rotterdam, Rotterdam 3015CE, the Netherlands; 26 Centre for Rare Diseases, University of Tübingen, 72072 Tübingen, Germany; 27 Institute for Ophthalmic Research, Center for Ophthalmology, University of Tübingen, 72076 Tübingen, Germany; 28 NIHR Biomedical Research Centre, Moorfields Eye Hospital, London EC1V 9EL, UK; 29 Manchester Centre for Genomic Medicine, Manchester University NHS Foundation Trust, Saint Mary’s Hospital, Manchester, UK; 30 Division of Evolution, Infection and Genomics, School of Biological Sciences, Faculty of Biology, Medicine and Health, University of Manchester, Manchester M13 9PL, UK; 31 Ophthalmology Service, Institute of Clinical Neurosciences of Southern Switzerland (INSI), Ente Ospedaliero Cantonale (EOC), Lugano, Switzerland; 32 Faculty of Biomedical Sciences, University of Southern Switzerland (USI), Lugano, Switzerland; 33 Department of Pathology & Cell Biology, Columbia University, New York, NY 10027, USA; 34 Department of Genetics and Genome Biology, University of Leicester, Leicester LE1 7RH, UK; 35 The Ruth & Bruce Rappaport Faculty of Medicine, Technion-Israel Institute of Technology, Haifa 31096, Israel; 36 Department of Ophthalmology, University of Leipzig, 04109 Leipzig, Germany

**Keywords:** macular dystrophy, cone-rod dystrophy, inherited retinal disease, hearing loss, *AP5B1*, fifth adaptor protein complex

## Abstract

Inherited retinal diseases (IRDs) represent a large group of genetically heterogeneous disorders that often cause progressive visual loss. The fifth adaptor protein (AP-5) complex, which contributes to endolysosomal trafficking and lysosomal homeostasis, has previously been implicated in neurodegenerative syndromes, and more recently, bi-allelic variants in three of its subunits were found in families with macular dystrophy, including four with variants in *AP5B1*. Here, we describe 22 affected individuals from 20 families with *AP5B1*-associated IRD, all carrying the recurrent missense variant c.2354T>C (GenBank: NM_138368.5) (p.Leu785Pro), either in the homozygous state (16 families) or in *trans* with another rare heterozygous *AP5B1* missense or loss-of-function variant. Five families were of Ashkenazi Jewish ancestry and 15 families of European ancestry. Clinically, affected individuals presented with a predominantly late-onset macular dystrophy that frequently progressed to cone-rod degeneration. Characteristic retinal findings included foveal sparing, early peripapillary involvement, and a reticular pattern best observed by fundus autofluorescence imaging in the mid- or peripheral retina. The typical presenting symptom was decreased visual acuity. Age at onset ranged from 27 to 74 years, with most individuals becoming symptomatic after the fifth decade of life. Some individuals also presented with extraocular manifestations, most notably hearing loss, which was reported in 8 affected individuals. These findings further support *AP5B1* as a cause of macular dystrophy, identify p.Leu785Pro as a relatively frequent pathogenic allele in individuals of European and Ashkenazi Jewish ancestry, and expand the associated phenotypic spectrum to include both isolated macular dystrophy and possible syndromic presentations.

## Teaser

This international study identifies the recurrent *AP5B1* c.2354T>C (p.Leu785Pro) variant as a relatively frequent cause of late-onset macular dystrophy in individuals of European and Ashkenazi Jewish ancestry. It defines characteristic retinal features, supports a founder effect, and broadens the phenotypic spectrum to include possible extraocular manifestations, particularly hearing loss.

## Introduction

Inherited retinal diseases (IRDs) comprise a clinically and genetically heterogeneous group of disorders characterized by progressive degeneration of photoreceptors and/or the retinal pigment epithelium (RPE) and represent a major cause of irreversible visual impairment worldwide.^1^ To date, pathogenic variants in more than 500 genes have been implicated in IRDs, highlighting the importance of diverse cellular pathways for retinal homeostasis maintenance and photoreceptor survival.^2^ Macular dystrophy represents a subgroup of IRDs primarily affecting the central retina and has been associated with variants in 31 genes according to RetiGene (v.1.13).

Among the pathways implicated in IRDs, intracellular vesicular trafficking and lysosomal maintenance have emerged as important mechanisms underlying both syndromic and non-syndromic IRDs. Some of these genes, including *CLN5* (MIM: 608102) and *MFSD8* (MIM: 611124), are also associated with neuronal ceroid lipofuscinosis, a group of lysosomal storage disorders, emphasizing the importance of endolysosomal pathways in retinal degeneration. The fifth adaptor protein (AP-5) complex mediates protein sorting within late endosomal trafficking pathways and contributes to lysosomal homeostasis.^3^ Structurally, AP-5 is a heterotetramer composed of four subunits, zeta, mu, beta, and sigma, encoded by *AP5Z1* (MIM: 613653), *AP5M1* (MIM: 614368), *AP5B1* (MIM: 614367), and *AP5S1* (MIM: 614824), respectively.^3^

Loss of *AP5Z1* was initially associated with hereditary spastic paraplegia (SPG48; MIM: 613647) accompanied by neuropathy, parkinsonism, and cognitive impairment.^4^^,^^5^^,^^6^ More recently, variants in *AP5Z1*, *AP5M1*, and *AP5B1* were implicated in recessive macular dystrophy in members of 19 families, suggesting an important role of AP-5-mediated trafficking in retinal function. Affected individuals exhibited a characteristic late-onset macular dystrophy with chorioretinal atrophy, peripapillary involvement, reticular pigmentary changes, and outer retinal tubulations. In that study, only two families carried bi-allelic variants in *AP5B1*, both involving loss-of-function (LoF) alleles.^7^ Hussain et al. independently reported additional individuals with *AP5Z1*- and *AP5B1*-associated macular dystrophy, including two patients carrying the recurrent *AP5B1* p.Leu785Pro (c.2354T>C) variant, further supporting the role of AP-5 complex dysfunction in IRD.^8^

Here, we report 20 additional families with *AP5B1*-associated IRD and further delineate the associated phenotypic spectrum. All families included at least one affected individual carrying the recurrent missense variant c.2354T>C (GenBank: NM_138368.5) (p.Leu785Pro), either in the homozygous state or in *trans* with another rare heterozygous *AP5B1* variant. Our findings substantially expand the clinical and genetic spectrum associated with *AP5B1*, identify p.Leu785Pro as a relatively frequent pathogenic allele in individuals of European and Ashkenazi Jewish ancestry, and further support an important role for AP-5 dysfunction in macular dystrophy.

## Material and methods

### Families and DNA samples

The study was conducted in accordance with the principles of the Declaration of Helsinki and received approval from the ethics committees of the participating institutions: Ethikkommission Nordwest-und Zentralschweiz; the Ethics Committee of the Medical Faculty, Eberhard Karls University Tübingen (project no. 116/2015BO2); Medical Ethics Review Committees of Tel Aviv Sourasky Medical Center, Bnai Zion Medical Center, Schneider Children’s Medical Center of Israel and Rambam Healthcare Campus; Ethics Committee of the Radboud University Medical Center (Nijmegen, the Netherlands); Rotterdam Eye Hospital (Rotterdam, the Netherlands) (MEC-2010-359; OZR protocol no. 2009-32); Columbia University (IRB-AAAI9906); Ethics Committee of the General University Hospital in Prague (reference no. 34/19); and Medical Ethics Committee of the Ghent University Hospital and East of England Cambridge South institutional review board (13/EE/0325).

Written informed consent was obtained from all individuals prior to their inclusion in this study.

### Clinical examination

Available clinical records from ophthalmic examinations were reviewed, including slit-lamp findings, Snellen best-corrected visual acuity (BCVA; converted to decimal values), and visual field testing performed using either Humphrey instruments (Carl Zeiss Meditec AG, Jena, Germany) or the Medmont M700 perimeter (Medmont International, Nunawading, Australia). Electrophysiological recordings were conducted using a Diagnosys system (Diagnosys, Lowell, MA, USA) in accordance with the standards of the International Society for Clinical Electrophysiology of Vision (ISCEV).^9^^,^^10^ Retinal imaging included wide-field color fundus photography and fundus autofluorescence (FAF), obtained using either the Clarus 700 system (Carl Zeiss Meditec AG) or Topcon cameras (Topcon Corporation, Tokyo, Japan), together with spectral-domain optical coherence tomography (SD-OCT) acquired using the Spectralis system (Heidelberg Engineering GmbH, Heidelberg, Germany).

### Molecular genetic analysis

DNA was obtained from venous blood or saliva samples. The initial screening cohort comprised 194 genetically unsolved unrelated individuals with macular dystrophy or related phenotypes, including cone-rod dystrophy and Stargardt disease, in whom prior routine diagnostic testing had not identified a causative variant. Exome (F1-II:1,^11^ F2-II:1,^12^ [F3-II:1, F4-II:1, F5-II:1],^13^ and [F17-II:1, F18-II:1, and F19-II:1]^14^) or genome ([F9-II:1, F10-II:1, F11-II:1, and F12-II:1],^15^ F14-II:1,^16^ and F20-II:1^17^) sequencing was carried out according to protocols specific to each participating institution. For F8-II:1, libraries were prepared and enriched using the KAPA HyperExome kit (Roche Sequencing Solutions, Pleasanton, CA, USA). For F13-II:1, exome sequencing was performed using the Twist Library Preparation EF Kit 2.0 and Twist Universal Adapter System (Twist Bioscience, South San Francisco, CA, USA). Exome libraries for F15-II:1 and F16-II:1 were prepared and sequenced by Psomagen (Rockville, MD, USA). For F6-II:1 and F7-II:1, *AP5B1* variants were identified by Sanger sequencing. Copy-number variation analysis in the homozygous affected individuals did not provide any indication of loss of heterozygosity due to large deletions by different methods.

Variant nomenclature was checked using VariantValidator,^18^ and variants were described according to Human Genome Variation Society (HGVS) recommendations.^19^ Variants were classified according to the American College of Medical Genetics and Genomics and the Association for Molecular Pathology (ACMG/AMP) guidelines.^20^ Their presence was assessed in the ClinVar database.^21^ Allele frequencies across different populations were obtained from gnomAD v.4.1.0,^22^ excluding UK Biobank (UKB) samples (non-UKB) to minimize overrepresentation of individuals with British ancestry, and from the All of Us database.^23^ Czech population-specific allele frequencies were obtained from the National Centre for Medical Genomics database (https://ncmg.cz). For deleteriousness predictors, we used VEST4^24^ and CADD,^25^ as REVEL scores are unavailable for *AP5B1* beyond amino acid position 327. Regions of homozygosity (ROHs) were assessed using AutoMap^26^ with default parameters. For F17-II:4, PLINK was used to assess ROHs.^27^

Where possible, identified variants were confirmed by Sanger sequencing, which was also used for segregation analysis in available first-degree relatives for three families. For F11-II:1, allelic cloning was performed following PCR amplification of a 1,769 bp fragment using primers 5′-CCTTACAGCACGAGAGCACA-3′ and 5′-GCAGCAGCAGCTTTGAGTC-3′, followed by cloning with the CloneJET PCR Cloning Kit (Thermo Fisher). A total of 38 clones were screened, of which 12 clones containing the expected insert were identified by colony PCR and subjected to Sanger sequencing. Four clones carried the c.892del (p.Arg298GlyfsTer19) variant, whereas eight clones carried the c.2354T>C variant, confirming that the two variants were located in *trans*. For F8-II:1 (c.1631T>C [p.Leu544Pro]), ONT long-read sequencing was performed to determine the phase of the variants following PCR amplification of a 1,196 bp fragment using primers 5′-ACTTTGTGGACCTCTTGGATCAG-3′ and 5′-AGCTACAGGAGTGTGCCTTG-3′. For F18-II:1 (c.2569C>T [p.Arg857Trp]), the two variants were confirmed to be in *trans* using a targeted long-read sequencing approach on PacBio Sequel IIe (Pacific Biosciences), as previously described.^28^ A 532-nt target region was first PCR amplified, and 90 ng of the pooled amplicon was used for library preparation. For F13-II:1, the two *AP5B1* variants were found in *trans* by investigating sequencing reads in IGV.

## Results

### Identification of a recurrent variant in *AP5B1*

**Figure 1:**
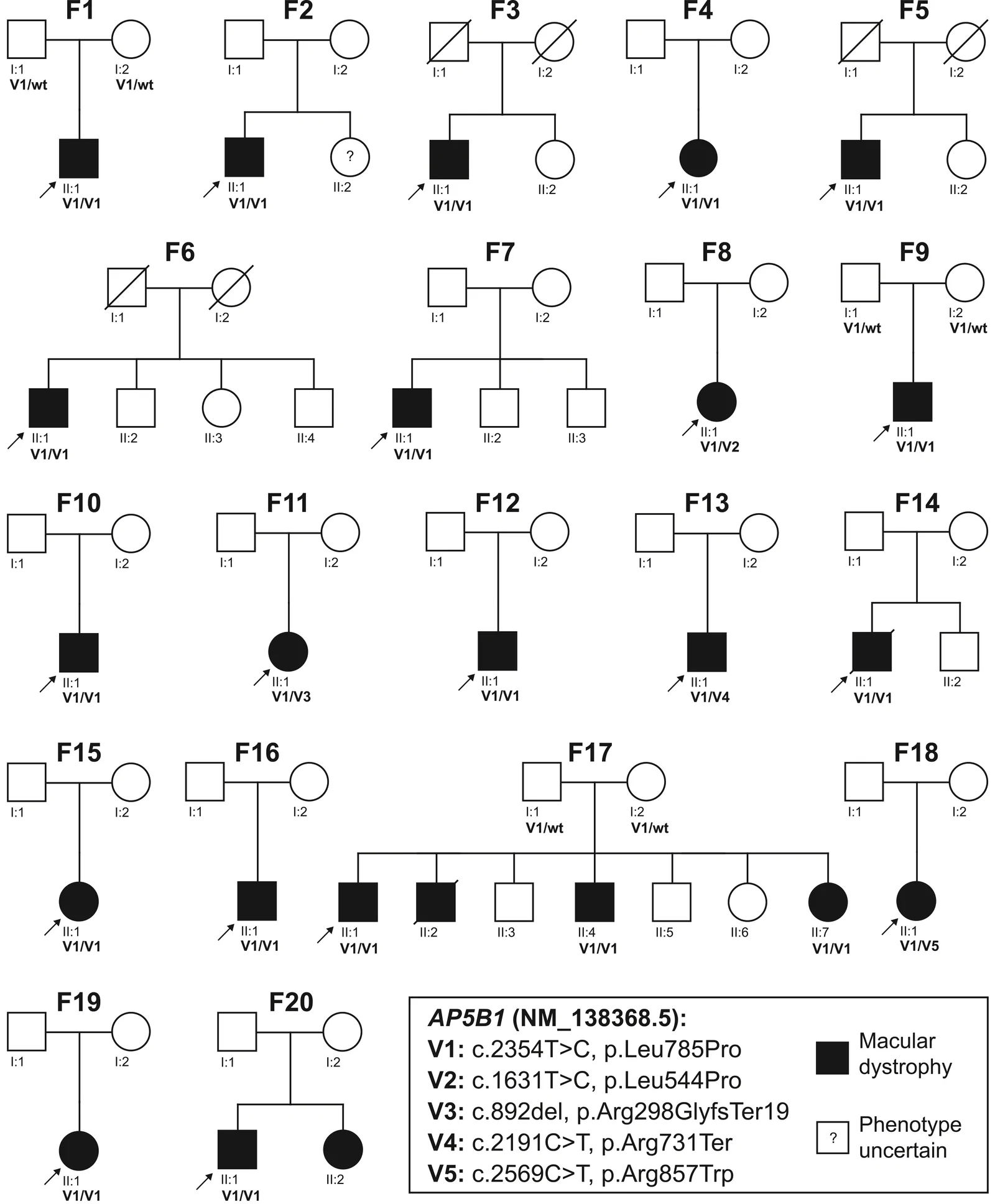
Pedigrees of 20 families with *AP5B1*-associated retinal dystrophy Variants are indicated according to GenBank: NM_138368.5. Filled shapes indicate affected individuals and unfilled shapes indicate unaffected individuals. Squares represent males and circles represent females. Diagonal lines represent deceased individuals. Genotypes are shown below each available individual, and genotypes in square brackets indicate that the phase of the variants is unknown.

Following the report by Kaminska et al. linking bi-allelic LoF alleles in *AP5B1* to macular dystrophy^7^ (Table S1), we investigated additional variants in this gene in individuals with genetically unsolved IRDs. Through the analysis of recurrent variants in a cohort of 194 individuals with genetically unsolved macular dystrophy or related phenotypes, including cone-rod dystrophy and Stargardt disease, we identified the recurrent missense variant in *AP5B1*, c.2354T>C (GenBank: NM_138368.5) (p.Leu785Pro), which was present in the homozygous state in three unrelated individuals from the Czech Republic (F1-II:1), Switzerland (F2-II:1), and Israel (F3-II:1) (Figure 1). In this initial cohort of 194 individuals with unsolved IRDs, predominantly of European ancestry as inferred using EthSEQ,^29^ the allele frequency of p.Leu785Pro was 1.55% (6 alleles). This represented a significant enrichment compared to gnomAD v.4.1.0 non-UKB non-Finnish European (NFE) populations, with an odds ratio of 11.9 (95% confidence interval [CI]: 4.31–26.2, Fisher’s exact test, *p* = 1.64 × 10^−5^).

The variant was located within ROHs spanning 4.55, 1.63, and 5.17 Mb, respectively (Figure S1). In addition, all three individuals shared three common homozygous *AP5B1* variants: c.509G>T (p.Gly170Val), c.631C>T (p.Leu211Phe), and c.1107T>G (p.Leu369=). Although these variants are too frequent in population databases to represent pathogenic recessive alleles (allele frequency of 12%–48% in gnomAD v.4.1.0 non-UKB populations), their co-occurrence with p.Leu785Pro suggested a shared ancestral haplotype and supported the hypothesis of a founder effect.

**Figure 2:**
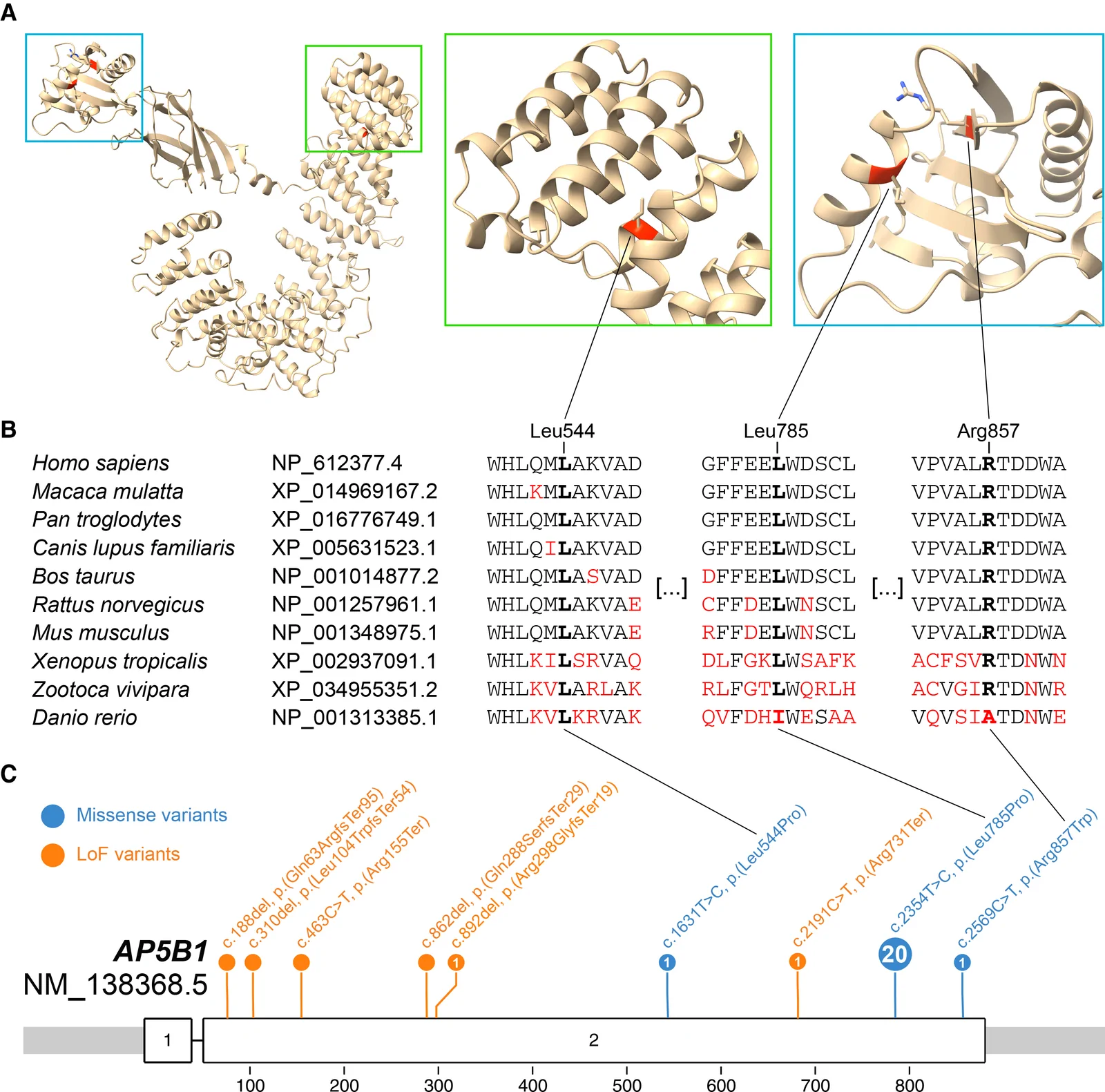
Genetic and protein-level overview of *AP5B1* variants associated with retinal dystrophy (A) Predicted structure of the AP5B1 protein (AlphaFold: AF-Q2VPB7-F1) highlighting the positions of the three missense variants identified in the current study: p.Leu544Pro, p.Leu785Pro, and p.Arg857Trp (shown in red). Insets show the local structural environment surrounding each altered residue. (B) Multispecies amino acid sequence alignment demonstrating evolutionary conservation of residues Leu544, Leu785, and Arg857 across selected vertebrate species. (C) Schematic representation of *AP5B1* (GenBank: NM_138368.5) with the distribution of variants associated with inherited retinal disease. Loss-of-function variants are shown in orange and missense variants in blue. Numbers in circles indicate the number of unrelated families carrying each variant in this study. Previously reported variants are also shown.

The p.Leu785Pro variant is classified in ClinVar^30^ as a variant of uncertain significance (VUS) and was previously reported in two individuals with macular dystrophy, one carrying the variant in the homozygous state and the other in *trans* with an *AP5B1* LoF variant. However, segregation analysis was not performed in either family.^8^ The Leu785 residue is highly conserved across mammals, amphibians, and reptiles (Figure 2). It is predicted to be deleterious by multiple in silico predictors with a VEST4 score^24^ of 0.92 and a CADD score^25^ of 25.2.

The allele frequency of c.2354T>C in the general population was relatively high for a pathogenic variant in an IRD-associated gene: 0.089% in gnomAD v.4.1.0 (non-UKB). The variant is most prevalent among individuals of Ashkenazi Jewish (0.36%) and non-Finnish European (NFE, 0.132%) ancestry while occurring at a lower frequency in Africans (0.0135%) and being absent from South and East Asian populations. Notably, one homozygous individual of NFE ancestry was present in gnomAD v.4.1.0. Allele frequencies were comparable in the All of Us database, with an overall frequency of 0.085% and 0.14% among NFE individuals. The variant was identified in 21 out of 3,598 alleles in the Czech population, corresponding to an allele frequency of 0.58%. Assuming Hardy-Weinberg equilibrium, these frequencies would predict homozygous individuals at an estimated prevalence of approximately 1:80,000 in the Ashkenazi Jewish population and 1:600,000 in Europeans overall, potentially reaching 1:30,000 in the Czech population.

Analysis of gnomAD v.2.1.1 subpopulation data further demonstrated marked intra-European variability, with higher allele frequencies observed in Eastern European populations, including Bulgarian (0.47%) and Estonian (0.37%) populations, compared to Northwestern European (0.060%) and South European (0.026%) populations. These findings support the hypothesis of a regional founder effect underlying the enrichment of p.Leu785Pro in specific European subpopulations.

Additional screening and reanalysis of IRD cohorts from the Czech Republic, Israel, the Netherlands, the UK, the USA, Germany, and Belgium identified an additional 19 affected individuals from 17 families. Thirteen families (15 affected individuals) carried c.2354T>C in the homozygous state, whereas in four families, this variant was found in the heterozygous state with an additional LoF or rare *AP5B1* missense variant: c.1631T>C (p.Leu544Pro), c.892del (p.Arg298GlyfsTer19), c.2191C>T (p.Arg731Ter), and c.2569C>T (p.Arg857Trp), each identified in a single family (Figure 1). These additional variants were very rare or absent from gnomAD v.4.1.0, with no homozygous individuals identified, and were all found in *trans* with c.2354T>C as established by allelic cloning, Oxford Nanopore Technologies or PacBio long-read sequencing, or read-backed phasing of short-read sequencing data (details in the material and methods). Available in silico predictions supported a deleterious effect for the missense variants (Table S1).

Furthermore, all 11 homozygous individuals for whom additional *AP5B1* variant information was available from exome/genome data carried the same three common homozygous *AP5B1* variants identified in the initial probands, and for ten of them, the variant was found in an ROH (Figure S1; Table S2). In addition, the three common *AP5B1* variants were present in the heterozygous state in three compound-heterozygous individuals for whom genotype data at these positions were available. Although their phase relative to p.Leu785Pro was not established, this recurrent co-occurrence is compatible with a shared ancestral background. Together with the homozygosity data and population-specific enrichment of p.Leu785Pro, these findings support the possibility of a founder effect. None of the 20 families included in the current study reported consanguinity.

Although the frequency of p.Leu785Pro in the NFE population is 0.132%, which is relatively high for a pathogenic variant associated with autosomal-recessive IRDs, it remains within the range reported for established pathogenic variants in other genes associated with autosomal-recessive IRD phenotypes. Examples include p.Cys759Phe (GenBank: NM_206933.4) (c.2276G>T) in *USH2A*, p.Thr383IlefsTer13 (GenBank: NM_019098.5) (c.1148del) in *CNGB3*, p.Met390Arg (GenBank: NM_024649.5) (c.1169T>G) in *BBS1*, p.Gly1961Glu (GenBank: NM_000350.3) (c.5882G>A) in *ABCA4*, and p.Pro261= (GenBank: NM_033100.4) (c.783G>A) in *CDHR1*, which show even higher allele frequencies in gnomAD v.4.1.0 non-UKB NFE populations (Table S3).

Given that the AP-5 complex is composed of four subunits, encoded by *AP5B1*, *AP5M1*, *AP5S1*, and *AP5Z1*, and based on previous reports of triallelism in IRDs,^31^^,^^32^ we also investigated the presence of additional variants in *AP5M1*, *AP5S1*, and *AP5Z1* using available exome and genome sequencing data. The three initial probands, homozygous for p.Leu785Pro, as well as eight other individuals for whom genetic data were available, did not carry additional rare (allele frequency of less than 1% in gnomAD non-UKB v.4.1.0) and potentially pathogenic variants in any of the queried genes, except for two individuals. F9-II:1 and F11-II:1 harbored variants meeting the filtering criteria: c.2105_2116dup (GenBank: NM_014855.3) (p.Met702_Leu705dup) in *AP5Z1* and c.599G>A (GenBank: NM_018347.3) (p.Arg200His) in *AP5S1*, respectively. These findings seem to argue against triallelism, but there are insufficient data to draw a firm conclusion.

### Phenotype associated with AP5B1

A total of 22 affected individuals (7 females) from 20 families carrying bi-allelic *AP5B1* genotypes consistent with recessive disease were included in the clinical analysis. Detailed demographic, clinical, and genetic data of the study cohort are summarized in Table S2.

All but one affected individual who underwent ophthalmic evaluation had clinical findings consistent with macular dystrophy, compatible with the previously described *AP5B1*-associated macular dystrophy phenotype.^7^ The most common presenting symptom was decreased BCVA reported in 15 individuals, followed by nyctalopia, which was present in six affected individuals. Additional symptoms included glare sensitivity and metamorphopsia. Information regarding age at symptom onset was available for 19 individuals. Based explicitly on reported ages, it ranged from 27 to 74 years (mean of 52.3 years and median of 53 years). One individual (F6-II:1) presented with nyctalopia and visual field constriction at the age of 57. Although retinal imaging was not available, full-field ERG findings documented in the patient’s records showed moderately reduced scotopic responses with milder photopic involvement, consistent with a diagnosis of rod-cone dystrophy.

**Figure 3:**
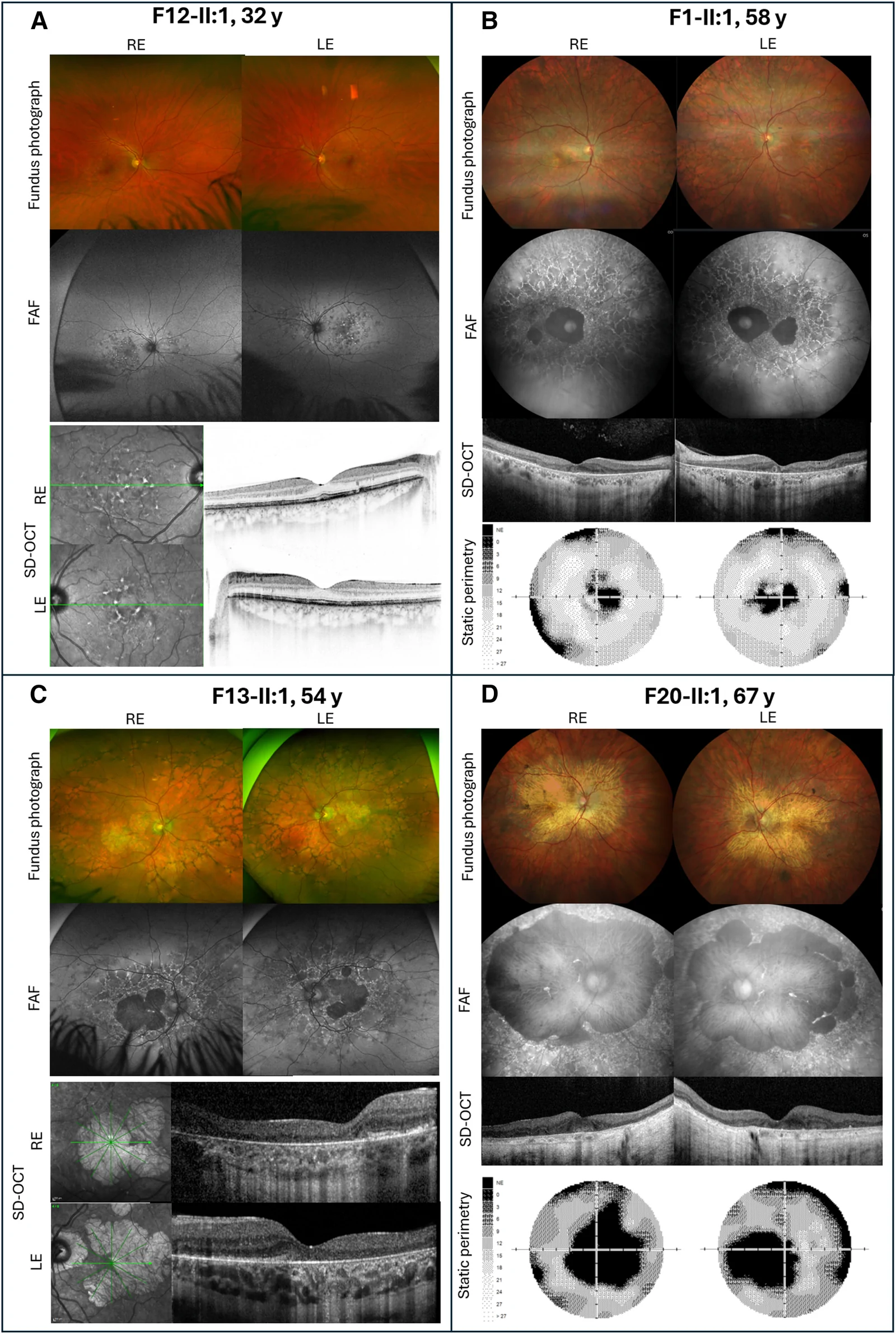
Phenotypic hallmarks of *AP5B1*-associated retinal dystrophy Four representative patients with disease stages ranging from mild to advanced are shown. The imaging modalities used, including fundus photography, fundus autofluorescence (FAF), spectral-domain optical coherence tomography (SD-OCT), and static perimetry (50° visual field), are indicated on the side for each patient. Comparison of the right eye (RE) and left eye (LE) demonstrates relatively symmetrical changes. (A) Individual F12-II:1 aged 32 years with mild disease showing irregularities of the retinal pigment epithelium predominantly in the macular region, with additional lesions surrounding the optic disc. (B) Individual F1-II:1 aged 58 years with moderately advanced disease. Note the areas of complete chorioretinal atrophy surrounding the optic discs and involving the macular region. The remaining posterior pole also demonstrates atrophic changes accompanied by a hyperautofluorescent reticular pattern. The pronounced atrophic lesions correspond to central scotomas detected by static perimetry. (C) Individual F13-II:1 with advanced disease characterized by extensive areas of complete atrophy in both the peripapillary and macular regions. Note the hyperautofluorescent reticular pattern surrounding these lesions. SD-OCT revealed outer retinal atrophy with relative foveal sparing in the LE. (D) Individual F20-II:1 with the most advanced disease stage, characterized by extensive complete atrophy throughout the posterior pole, corresponding to a large central scotoma and severe outer retinal atrophy on SD-OCT.

Fundus findings in the early stages included peripapillary atrophy and patchy, typically oval-shaped areas of chorioretinal atrophy within the posterior pole, showing corresponding hypoautofluorescence on FAF imaging and usually involving the macular and peripapillary regions (Figures 3A and 3B). In advanced stages, the atrophic areas progressively enlarged to encompass the entire posterior pole and extended beyond the vascular arcades (Figures 3C and 3D). Surrounding the atrophic regions, predominantly in the mid-peripheral and peripheral retina, a reticular pattern was observed on FAF imaging and/or by slit-lamp examination (Figures 3B and 3C). Corresponding to the central atrophic lesions, patients exhibited reduced BCVA and central scotomas on visual field testing (Figures 3B and 3D).

In general, visual function tended to decline with increasing age and disease duration, although the rate and severity of progression varied considerably among affected individuals. Patients in the early stages of the disease or shortly after symptom onset often retained relatively preserved BCVA, whereas older individuals and those with a longer history of symptoms more frequently exhibited advanced visual loss. Notably, several individuals in their sixth to eighth decades demonstrated profound bilateral visual impairment with BCVA in the range of hand movement, while some younger or mildly affected patients maintained near-normal visual acuity despite the presence of retinal abnormalities. Marked interocular asymmetry in BCVA measurements was also observed in a subset of affected individuals. This was attributed to foveal sparing. In general, foveal sparing in one or both eyes was present in 12 individuals and was considered one of the typical features of the *AP5B1-*associated retinal phenotype (Figure 3C).

Optic discs appeared normal without pallor, and vessel attenuation was only observed in advanced stages (Figures 3C and 3D). Full-field ERG findings were normal in the early stages of the disease; however, as the disease progressed, both photopic and scotopic responses became variably reduced, consistent with either cone-rod or rod-cone dysfunction. Multifocal ERG documented initial cone alteration in one affected individual. Although several patients exhibited mild to moderate refractive errors, these were considered to fall within the range expected in the general population.

Some affected individuals also exhibited extraocular features; however, most were considered age-related and/or independent comorbidities, including diabetes mellitus, hypertension, hyperlipidemia, borderline renal dysfunction, benign prostatic hyperplasia, chronic insomnia, tinnitus, panic attacks, depression, breast cancer, and thyroid dysfunction. One patient had developed peripheral neuropathy at the age of 42. Hearing loss was reported in eight patients (36% in our cohort) and was considered a potentially relevant associated feature. However, audiometric data were not available, and systematic screening for pathogenic variants in genes commonly associated with hereditary hearing loss could not be confirmed across the participating centers. No alternative molecular diagnosis accounting for the hearing impairment was reported to us by the collaborating centers. Nevertheless, additional genetic factors contributing to hearing loss cannot be excluded. It should also be noted that asymptomatic individuals were not systematically evaluated for extraocular manifestations, raising the possibility that additional or mild features were not recognized.

## Discussion

This multicenter study describes 22 individuals from 20 families with *AP5B1*-associated IRD, including individuals homozygous for p.Leu785Pro and individuals carrying p.Leu785Pro in *trans* with another heterozygous rare *AP5B1* variant. Some affected individuals had extraocular features, most notably hearing loss. Four individuals with bi-allelic *AP5B1* variants and IRD had previously been reported.^7^^,^^8^ Therefore, our findings firmly establish *AP5B1* as a disease-causing gene for late-onset macular dystrophy and suggest that some affected individuals may have extraocular manifestations.

We identified five distinct *AP5B1* variants, including two LoF and three missense variants, of which four were not previously reported for a macular phenotype, further highlighting the allelic heterogeneity associated with this gene. Notably, all affected individuals carried the recurrent p.Leu785Pro variant, either in the homozygous state or in *trans* with another rare heterozygous *AP5B1* variant, supporting its pathogenic role.

The precise molecular effect of p.Leu785Pro remains unknown. Leu785 is evolutionarily conserved and lies within a structured region of the predicted AP5B1 protein model. The substitution of leucine with proline could perturb local secondary structure because of the conformational constraints imposed by proline, potentially affecting AP5B1 stability, incorporation into the heterotetrameric AP-5 complex, or interactions with other AP-5 components. However, these possibilities remain speculative because variant-specific functional studies have not been performed. More generally, loss of AP5B1 or AP5Z1 has been associated with reduced AP5M1 abundance, consistent with destabilization of an incompletely assembled AP-5 complex, while depletion of AP5B1 or AP5M1 alters the localization of lysosomal membrane and retromer proteins and increases the size of multivesicular bodies near the Golgi apparatus.^3^^,^^4^ Kaminska et al. demonstrated punctate AP5B1 staining in human RPE explants and induced pluripotent stem cell (iPSC)-derived RPE cells, with the strongest co-localization observed with the late-endosomal marker Rab7 and partial overlap with TGN46-positive Golgi-derived vesicles.^7^ Together, these findings make impaired endolysosomal protein sorting and lysosomal homeostasis a plausible consequence of reduced AP5B1 function. Nevertheless, single-cell RNA sequencing data summarized in RetiGene do not indicate marked enrichment of *AP5B1* in any specific retinal cell type, suggesting that its retinal function may not be restricted to the RPE.^2^ Functional studies assessing mutant protein abundance, AP-5 complex assembly, partner interactions, and endolysosomal trafficking will be required to establish the mechanism of p.Leu785Pro.

The ocular phenotype associated with bi-allelic variants in *AP5B1* is a progressive macular dystrophy in which structural retinal abnormalities may precede symptomatic visual loss. Longitudinal data available for seven individuals demonstrated progressive, largely symmetrical retinal degeneration. In our cohort, symptomatic onset was predominantly late: the reported age at symptom onset ranged from 27 to 74 years, with a median of 53 years, and most individuals became symptomatic after 50 years of age. Even among patients aged 50 years or older, 25 of 32 eyes had no or mild visual impairment according to WHO categories, whereas 7 of 32 eyes had moderate or more severe visual impairment. Early stages are characterized by subtle abnormalities affecting predominantly the macular region, often with additional peripapillary involvement and rod dysfunction. Multiple small hyperautofluorescent lesions were observed in some individuals, including fleck-like lesions at the posterior pole and nasal to the optic disc in F12-II:1 and focal lesions along the margin of a large hypoautofluorescent area at the posterior pole in F10-II:1. These findings prompted initial suspected diagnoses of Stargardt disease or pattern dystrophy. As the disease progresses, areas of complete chorioretinal atrophy develop in the macular and peripapillary regions and gradually expand. These changes are accompanied by a reticular pattern corresponding to hyperautofluorescent lesions. In advanced stages, total chorioretinal atrophy involves much of the posterior pole, leading to large central scotomas, profound outer retinal degeneration, and severe visual impairment. Consistent with the predominantly late symptomatic onset, one patient was initially referred with suspected age-related macular degeneration. Recurrent features of *AP5B1*-associated disease included foveal sparing, early peripapillary involvement, and a reticular pattern adjacent to areas of profound chorioretinal atrophy.

The extraocular manifestations observed in several individuals raise the possibility of a broader *AP5B1*-associated phenotype, but they should be interpreted cautiously because systemic assessment was not standardized and affected individuals were not evaluated in the absence of relevant clinical symptoms. In addition, some findings may reflect age-related or independent comorbidity. Thus, the association of additional systemic manifestations requires further validation. Interestingly, one of the four previously described *AP5B1*-associated affected individuals was also noted to suffer from polyneuropathy and hearing loss.^7^^,^^8^ Hearing impairment, peripheral neuropathy, and renal involvement also overlap with manifestations reported in patients with *AP5Z1*-associated neurodegenerative disease, suggesting that dysfunction of different AP-5 subunits may produce a broader phenotypic spectrum,^7^ and these are biologically consistent with the known role of the AP-5 complex in intracellular trafficking and lysosomal homeostasis.^4^ Public transcriptomic resources currently provide no clear evidence that *AP5B1* is enriched in a specific auditory cell type. *AP5B1* shows broad but relatively low expression across tissues represented in GTEx. In the inner-ear datasets examined using gEAR, *AP5B1* was absent or detected at very low levels, without convincing enrichment in a particular cochlear cell population. Because low-abundance transcripts may not be consistently detected by single-cell RNA sequencing, these observations do not exclude biologically relevant expression in the inner ear. Nevertheless, the available data do not currently support a specific mechanism involving cochlear hair cells, spiral ganglion neurons, or another auditory cell population.

Although triallelism and oligogenic inheritance have been proposed in some IRDs, most notably Bardet-Biedl syndrome,^31^^,^^32^ their contribution to IRD pathogenesis remains incompletely understood. In our cohort, two affected individuals additionally carried rare variants in *AP5Z1* or *AP5S1*, raising the possibility that variants affecting multiple AP-5 complex subunits could potentially act as phenotypic modifiers. However, no clear evidence supporting clinically relevant triallelism was identified, and the limited clinical data available did not suggest a consistently more severe phenotype in these individuals compared with the remainder of the cohort. Further studies with larger cohorts and more detailed phenotyping will be required to clarify whether additional AP-5 complex variants may contribute to phenotypic variability in *AP5B1*-associated disease.

A limitation of this study is the heterogeneity of the clinical data, which were collected across eight major international centers, with additional contributions from affiliated national sites. While this broad collaboration strengthened the overall sample size and geographic representation, differences in documentation meant that certain parameters were not consistently recorded. Formal haplotype reconstruction and dating of the putative founder allele were also not performed because genome-wide data suitable for such analyses were not available for all individuals, and exome sequencing provides limited coverage for this purpose. Yet, the recurrent co-occurrence of p.Leu785Pro with shared homozygous *AP5B1* polymorphisms and identified ROHs further supports the hypothesis of a founder event. The markedly increased allele frequency observed in Eastern European and Ashkenazi Jewish (of Central and Eastern Europe origins) populations compared with other European subgroups is also compatible with regional founder enrichment.

The relatively high population frequency of c.2354T>C represents an unusual but not unprecedented finding for a recessive IRD-associated allele. Several established pathogenic IRD variants, particularly those associated with hypomorphic or late-onset phenotypes, show similarly elevated frequencies in population databases. The strong enrichment observed in our IRD cohort, the presence of multiple unrelated homozygous affected individuals (*n* = 18), segregation in one family with multiple affected individuals, and the occurrence of the variant in *trans* with independent LoF alleles (*n* = 4) collectively support the pathogenicity of p.Leu785Pro.

For *AP5B1*, the cumulative allele frequency of LoF variants fulfilling PVS1 ACMG criteria in gnomAD v.4.1.0 non-UKB NFE populations reaches 0.051%. Assuming Hardy-Weinberg equilibrium, this would predict approximately 1:750,000 individuals carrying p.Leu785Pro in *trans* with a LoF allele. When combined with the expected frequency of homozygous p.Leu785Pro individuals, the estimated prevalence of bi-allelic *AP5B1*-associated disease may reach approximately 1:325,000 in European populations. Given the large global population of European ancestry, this could theoretically correspond to several thousand affected individuals worldwide, suggesting that *AP5B1*-associated disease is likely substantially underdiagnosed. Similar calculations in the Ashkenazi Jewish population suggest a prevalence approaching 1:70,000 individuals. Importantly, population frequencies varied substantially across European subgroups. While the allele frequency reached 0.132% in NFE overall, markedly higher frequencies were observed in Eastern European populations, including Czech, Bulgarian, and Estonian populations, where the estimated prevalence of homozygous individuals may approach approximately 1:30,000 individuals. Since these estimates do not include other potentially pathogenic missense alleles, the true prevalence may be even higher.

The presence of homozygous individuals in population databases does not exclude pathogenicity, particularly for late-onset IRD with incomplete clinical ascertainment.^33^^,^^34^^,^^35^ Individuals included in population reference databases may still harbor genetic disease, especially when manifestations are mild, slowly progressive, or diagnosed late in life. This is in line with the fact that gnomAD v.4.1.0 contains one European individual homozygous for p.Leu785Pro for whom age information was unavailable, while gnomAD v.2.1.1 included two European individuals homozygous for p.Leu785Pro, aged between 50 and 65 years.

In summary, this international study provides strong evidence that *AP5B1* is a cause of late-onset macular dystrophy, identifies p.Leu785Pro as a relatively frequent pathogenic allele in patients of European and Ashkenazi Jewish origin, supports a common ancestral origin of this allele, and expands the associated spectrum to include IRDs with both cone and rod involvement and variable extraocular manifestations. Future prospective studies with standardized neurological and systemic evaluation will be essential to better define the systemic features associated with bi-allelic *AP5B1* variants.

## Data and code availability

The sequencing data generated and analyzed during the current study are not publicly available due to privacy constraints. However, specific variants can be obtained from the corresponding author upon reasonable request. All variants identified in this study have been submitted to the ClinVar database (SUB16335131).

## Acknowledgments

The authors thank all patients and their families for participating in this study; the National Center for Medical Genomics of the Czech Republic, Prague (LM2023067), for providing allele frequencies in an ethnically matched population; and the European Reference Network for Rare Eye Diseases (ERN-EYE), within which this work was generated (University Hospitals in Prague, Ghent, Tübingen, and Nijmegen). Support was provided by the Swiss RetinAward (M.Q.); 10.13039/501100001711Swiss National Science Foundation grant 176097 (C.R.); 10.13039/501100003977Israel Science Foundation grant 331/24 (T.B.-Y.); HORIZON-MSCA-2022-DN ProgRET grant 101120562 (to S.R., E.D.B., and S.K.; supporting S.S.); Algemene Nederlandse Vereniging ter voorkoming van Blindheid, Oogfonds, Landelijke Stichting voor Blinden en Slechtzienden, Rotterdamse Stichting Blindenbelangen, Stichting Blindenhulp, Stichting tot Verbetering van het Lot der Blinden, Stichting Blinden-Penning, Foundation Fighting Blindness Career Development Award CD-GE-0621-0809-RAD, Radboudumc Starter Grant OZI-23.009, and 10.13039/501100003246NWO Aspasia 015.021.028 (S.R.); 10.13039/100000002NIH grants R01 EY028203, R01 EY028954, R01 EY029315, R01 EY024091, R01 EY036061, and P30 EY019007 (Core Grant for Vision Research) and an unrestricted Research to Prevent Blindness, USA, grant to Columbia University’s Department of Ophthalmology (J.Z. and R.A.); 10.13039/100000002NIH grant K99 EY036930-02 and Stanley J. Glaser Foundation Award UM SJG 2026-06 (W.L.); Czech Ministry of Health grant NW24-06-00083, RVO VFN64165 and Charles University programs UNCE/24/MED/022 and SVV 2600631 (L.D., B.K., M.V., and P.L.); Medical Research Council Clinician Scientist Fellowship UKRI440 and National Institute for Health and Care Research (NIHR) Manchester Biomedical Research Centre (BRC) NIHR203308 (S.L.); and University of Tübingen Faculty of Medicine Clinician Scientist Program grant 538-0-0 (A.S.).

## Author contributions

M.Q. and P.L. conceived and coordinated the study. P.L., L.K., and A.S. collected and analyzed the clinical data. M.Q., P.M., T.B.H., and S.K. collected and analyzed the genetic data, performed the variant interpretation, conducted the population genetic analyses, and contributed to the interpretation of allele frequency and founder effect data. P.L., L.D., and M.Q. drafted the manuscript. P.L., L.K., L.D., B.K., M.V., R.L., M.E., D.S., S.L., A.R.W., L.I.v.d.B., C.C.W.K., W.L., Y.v.L., D.Z., F.Z., V.B., J.D.Z., B.W.,M.M., K.P., C.R., and A.S. contributed to clinical data and performed ocular phenotyping in affected individuals. L.D., M.Q., P.M., T.B.H., S.K., T.B.-Y., S.L., G.A., K.K., L.H.-W., S.S., S.R., J.Z., M.B., E.D.B., F.C., and K.P. performed the molecular genetic analyses, including exome/genome sequencing, segregation studies, and bioinformatic analyses of specific cases. R.A. supervised W.L. and J.Z. and obtained funding. All authors reviewed and edited the manuscript.

## Declaration of interests

The authors declare no competing interests.

## Declaration of generative AI and AI-assisted technologies in the writing process

During the preparation of this work, the authors used ChatGPT for review of the text and corrections. The authors reviewed and edited the output as needed and take full responsibility for the content of the published article.

## Web resources

ClinVar, https://www.ncbi.nlm.nih.gov/clinvar/
Czech National Centre for Medical Genomics database, https://ncmg.cz
gEAR, https://www.umgear.org/
GenBank, https://www.ncbi.nlm.nih.gov/genbank/
gnomAD, https://gnomad.broadinstitute.org/
GTEx, https://gtexportal.org/
OMIM, https://www.omim.org
RetiGene, https://retigene.erdc.info/
VariantValidator, https://variantvalidator.org/

## Supplemental information

Data S1. Tables S1–S3

Document S2. Article plus supplemental information
