# Supplementary material for: The AP5B1 p.Leu785Pro variant is a frequent cause of late-onset macular dystrophy with variable extraocular manifestations
Source: HGG Adv. 2026 Aug 7;7(4):100659. doi: 10.1016/j.xhgg.2026.100659 (PMC13524567; doi:10.1016/j.xhgg.2026.100659)
Supplement: Supplementary file 1 [file mmc1.pdf]

HGGA, Volume 7

Supplemental information

The *AP5B1* p.Leu785Pro variant is a frequent cause of late-onset macular dystrophy with variable extraocular manifestations

Petra Liskova, Lubica Dudakova, Karolina Kaminska, Laura Kühlewein, Stefanida Shliaga, Rina Leibu, Miriam Ehrenberg, Dinah Zur, Jana Zernant, Miriam Bauwens, Gavin Arno, Isabelle Müller, Focke Ziemssen, Viktoria Bothe, Francesca Cancellieri, Julie De Zaeytijd, Pascale Mazzola, Winston Lee, Bohdan Kousal, Marie Vajter, L. Ingeborgh van den Born, Anton Sonntag, Yoeri van Leeuwen, Dzenita Smailhodzic, Caroline C.W. Klaver, Tobias B. Haack, Bernd Wissinger, Andrew R. Webster, Lonneke Haer-Wigman, Siying Lin, Moreno Menghini, Konrad Platzer, Elfride De Baere, Rando Allikmets, Carlo Rivolta, Susanne Roosing, Susanne Kohl, Tamar Ben-Yosef, and Mathieu Quinodoz

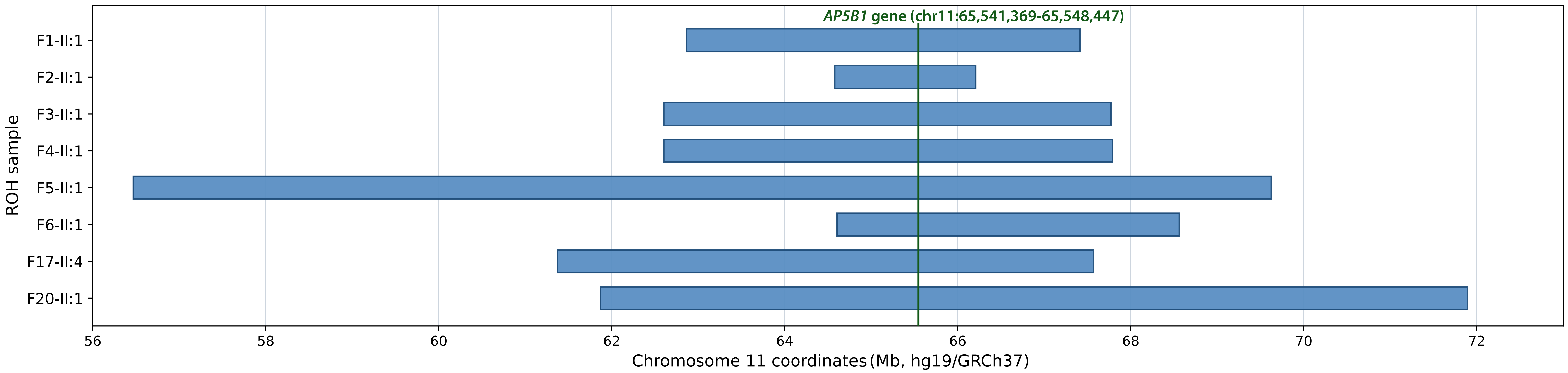

Figure S1: Homozygosity mapping at the *AP5B1* locus. Chromosome 11 regions of homozygosity (ROH) determined with AutoMap and PLINK and overlapping the *AP5B1* genomic region in the hg19/GRCh37 reference assembly. Each blue horizontal bar represents a ROH interval identified in one sample. The green bar indicates the genomic span of *AP5B1* at chr11:65,541,369-65,548,447. The NM_138368.5:c.2354T>C variant position, chr11:65,545,610, falls within the *AP5B1* locus and is contained within all ROH intervals.
